# Cohort Profile: Burden of Obstructive Lung Disease (BOLD) study

**DOI:** 10.1093/ije/dyad146

**Published:** 2023-10-20

**Authors:** Andre F S Amaral, James Potts, Ben Knox-Brown, Emmanouil Bagkeris, Imed Harrabi, Hamid Hacene Cherkaski, Dhiraj Agarwal, Sanjay Juvekar, Mahesh Padukudru Anand, Thorarinn Gislason, Asaad Ahmed Nafees, Kevin Mortimer, Christer Janson, Li Cher Loh, Stefanni Nonna Paraguas, Meriam Denguezli, Mohammed Al Ghobain, David Mannino, Martin W Njoroge, Graham Devereux, Terence Seemungal, Cristina Barbara, Ali Kocabaş, Rana Ahmed, Althea Aquart-Stewart, Michael Studnicka, Tobias Welte, Wan C Tan, Richard N van Zyl-Smit, Parvaiz Koul, Vanessa Garcia-Larsen, Cosetta Minelli, A Sonia Buist, Peter Burney, the BOLD Study Collaborative Network, Hasan Hafizi, Anila Aliko, Donika Bardhi, Holta Tafa, Natasha Thanasi, Arian Mezini, Alma Teferici, Dafina Todri, Jolanda Nikolla, Rezarta Kazasi, Hamid Hacene Cherkaski, Amira Bengrait, Tabarek Haddad, Ibtissem Zgaoula, Maamar Ghit, Abdelhamid Roubhia, Soumaya Boudra, Feryal Atoui, Randa Yakoubi, Rachid Benali, Abdelghani Bencheikh, Nadia Ait-Khaled, Christine Jenkins, Guy Marks, Tessa Bird, Paola Espinel, Kate Hardaker, Brett Toelle, Michael Studnicka, Torkil Dawes, Bernd Lamprecht, Lea Schirhofer, Herve Lawin, Arsene Kpangon, Karl Kpossou, Gildas Agodokpessi, Paul Ayelo, Benjamin Fayomi, Rolus Atrokpo, Gaston Hounton, Dieudonnè Yadjodo, Bertrand Mbatchou, Atongno Humphrey Ashu, Wan C Tan, Wen Wang, NanShan Zhong, Shengming Liu, Jiachun Lu, Pixin Ran, Dali Wang, Jin-ping Zheng, Yumin Zhou, Rain Jõgi, Hendrik Laja, Katrin Ulst, Vappu Zobel, Toomas-Julius Lill, Katrin Kiili, Ira Laanelepp, Tobias Welte, Isabelle Bodemann, Henning Geldmacher, Alexandra Schweda-Linow, Thorarinn Gislason, Bryndis Benedikdtsdottir, Kristin Jörundsdottir, Lovisa Gudmundsdottir, Sigrun Gudmundsdottir, Gunnar Gudmundsson, Elin Helga Thorarinsdottir, Hjördis Sigrun Pálsdottir, Mahesh Padukudru Anand, Parvaiz A Koul, Sajjad Malik, Nissar A Hakim, Umar Hafiz Khan, Rohini Chowgule, Vasant Shetye, Jonelle Raphael, Rosel Almeda, Mahesh Tawde, Rafiq Tadvi, Sunil Katkar, Milind Kadam, Rupesh Dhanawade, Umesh Ghurup, Sanjay Juvekar, Siddhi Hirve, Somnath Sambhudas, Bharat Chaidhary, Meera Tambe, Savita Pingale, Arati Umap, Archana Umap, Nitin Shelar, Sampada Devchakke, Sharda Chaudhary, Suvarna Bondre, Savita Walke, Ashleshsa Gawhane, Anil Sapkal, Rupali Argade, Vijay Gaikwad, Dhiraj Agrawal, Babu Pawar, Shalan Mhetre, Namdev Kale, Shirish Kathale, Sundeep Salvi, Bill Brashier, Jyoti Londhe, Sapna Madas, Althea Aquart-Stewart, Akosua Francia Aikman, Talant M Sooronbaev, Bermet M Estebesova, Meerim Akmatalieva, Saadat Usenbaeva, Jypara Kydyrova, Eliza Bostonova, Ulan Sheraliev, Nuridin Marajapov, Nurgul Toktogulova, Berik Emilov, Toktogul Azilova, Gulnara Beishekeeva, Nasyikat Dononbaeva, Aijamal Tabyshova, Kevin Mortimer, Wezzie Nyapigoti, Ernest Mwangoka, Mayamiko Kambwili, Martha Chipeta, Gloria Banda, Suzgo Mkandawire, Justice Banda, Graham Devereux, Jamie Rylance, Martin Njoroge, Catherine Chirwa, Chifundo Mhango, Edgar Ngwira, Faith Zumazuma, Frank Jonas, Patrick Mjojo, Li-Cher Loh, Abdul Rashid, Siti Sholehah, Mohamed C Benjelloun, Chakib Nejjari, Mohamed Elbiaze, Karima El Rhazi, Manelle Rjimati, Btissame ElHarche, Reda Benjelloun, Yassin Chefchaou, E F M Wouters, G J Wesseling, Daniel Obaseki, Gregory Erhabor, Olayemi Awopeju, Olufemi Adewole, Amund Gulsvik, Tina Endresen, Lene Svendsen, Rune Nielsen, Marit Aardal, Hildegunn B Fleten, Gerd Eli Dale, Eli Nordeide, Malin P Grøttveit, Åsa Skjelde, Ane Aamli Gagnat, Anders Ørskov Rotevatn, Marta Erdal, Asaad A Nafees, Muhammad Irfan, Hasan Nawaz Tahir, Muhammad Noman, Roman Ul Haq, Luisito F Idolor, Teresita S de Guia, Norberto A Francisco, Camilo C Roa, Fernando G Ayuyao, Cecil Z Tady, Daniel T Tan, Sylvia Banal-Yang, Vincent M Balanag, Maria Teresita N Reyes, Renato B Dantes, Stefanni Nonna M Paraguas, Renato B Dantes, Lourdes Amarillo, Lakan U Berratio, Lenora C Fernandez, Norberto A Francisco, Gerard S Garcia, Teresita S de Guia, Luisito F Idolor, Sullian S Naval, Thessa Reyes, Camilo C Roa, Ma Flordeliza Sanchez, Leander P Simpao, Ewa Nizankowska-Mogilnicka, Jakub Frey, Rafal Harat, Filip Mejza, Pawel Nastalek, Andrzej Pajak, Wojciech Skucha, Andrzej Szczeklik, Magda Twardowska, Cristina Bárbara, Fátima Rodrigues, Hermínia Dias, João Cardoso, João Almeida, Maria João Matos, Paula Simão, Moutinho Santos, Reis Ferreira, M Al Ghobain, H Alorainy, E El-Hamad, M Al Hajjaj, A Hashi, R Dela, R Fanuncio, E Doloriel, I Marciano, L Safia, Eric Bateman, Anamika Jithoo, Desiree Adams, Edward Barnes, Jasper Freeman, Anton Hayes, Sipho Hlengwa, Christine Johannisen, Mariana Koopman, Innocentia Louw, Ina Ludick, Alta Olckers, Johanna Ryck, Janita Storbeck, Richard van Zyl-Smit, Kirthi Gunasekera, Rajitha Wickremasinghe, Asma Elsony, Hana A Elsadig, Nada Bakery Osman, Bandar Salah Noory, Monjda Awad Mohamed, Hasab Alrasoul Akasha Ahmed Osman, Namarig Moham ed Elhassan, Abdel Mu'is El Zain, Marwa Mohamed Mohamaden, Suhaiba Khalifa, Mahmoud Elhadi, Mohand Hassan, Dalia Abdelmonam, Rana Ahmed, Rashid Osman, Hind Eltigani, Najlaa Mohamed Abass, Ahmed Beriar Ahmed, Sahar AlaElddin, Christer Janson, Inga Sif Olafsdottir, Katarina Nisser, Ulrike Spetz-Nyström, Gunilla Hägg, Gun-Marie Lund, Andrei Malinovschi, Eva Wallberg, Birgitta Appelfeldt, Mona Andrén, Terence Seemungal, Fallon Lutchmansingh, Liane Conyette, Imed Harrabi, Myriam Denguezli, Zouhair Tabka, Hager Daldoul, Zaki Boukheroufa, Firas Chouikha, Wahbi Belhaj Khalifa, Safa Hsan, Nadia Lakhdar, Mounir Landolsi, Ali Kocabaş, Attila Hancioglu, Ismail Hanta, Sedat Kuleci, Ahmet Sinan Turkyilmaz, Sema Umut, Turgay Unalan, Peter G J Burney, Anamika Jithoo, Louisa Gnatiuc, Hadia Azar, Jaymini Patel, Caron Amor, James Potts, Michael Tumilty, Fiona McLean, Risha Dudhaiya, Andre F S Amaral, Octavia Mulhern, Emmanouil Bagkeris, Jasleen Gegic, Paul Cullinan, Cosetta Minelli, A Sonia Buist, Mary Ann McBurnie, William M Vollmer, Suzanne Gillespie, Sean Sullivan, Todd A Lee, Kevin B Weiss, Robert L Jensen, Robert Crapo, Paul Enright, David M Mannino, John Cain, Rebecca Copeland, Dana Hazen, Jennifer Methvin, Vanessa Garcia Larsen

**Affiliations:** Ntational Heart and Lung Instiute, Imperial College London, London, UK; NIHR Imperial Biomedical Research Centre, London, UK; Ntational Heart and Lung Instiute, Imperial College London, London, UK; Ntational Heart and Lung Instiute, Imperial College London, London, UK; Ntational Heart and Lung Instiute, Imperial College London, London, UK; Ibn El Jazzar Faculty of Medicine of Sousse, University of Sousse, Sousse, Tunisia; Department of Pneumology, Faculty of Medicine Annaba, University Badji Mokhtar of Annaba, Annaba, Algeria; Vadu Rural Health Program, KEM Hospital Research Centre, Pune, India; Vadu Rural Health Program, KEM Hospital Research Centre, Pune, India; Department of Respiratory Medicine, JSS Medical College, JSSAHER, Mysuru, India; Faculty of Medicine, University of Iceland, Reykjavík, Iceland; Department of Sleep, Landspitali—National University Hospital of Iceland, Reykjavík, Iceland; Department of Community Health Sciences, Aga Khan University, Karachi, Pakistan; University of Cambridge, Cambridge, UK; Liverpool University Hospitals NHS Foundation Trust, Liverpool, UK; Department of Medical Sciences: Respiratory, Allergy and Sleep Research, Uppsala University, Uppsala, Sweden; Royal College of Surgeons in Ireland and University College Dublin Malaysia Campus, Penang, Malaysia; Philippine College of Chest Physicians, Manila, Philippines; Laboratoire de Recherche en Physiologie de l’Exercice et Physiopathologie, de l’Intégré au Moleculaire (LR19ES09), Faculté de Médecine de Sousse, Université de Sousse, Sousse, Tunisia; King Saud bin Abdulaziz, University for Health Sciences, Riyadh, Saudi Arabia; King Abdullah International Medical Research Centre, Riyadh, Saudi Arabia; University of Kentucky, Lexington, KY, USA; COPD Foundation, Miami, FL, USA; Centre for Reviews and Dissemination, University of York, York, UK; Department of Clinical Sciences, Liverpool School of Tropical Medicine, Liverpool, UK; Department of Clinical Sciences, Liverpool School of Tropical Medicine, Liverpool, UK; University of The West Indies, St Augustine Campus, St Augustine, Trinidad and Tobago; Institute of Environmental Health, Lisbon Medical School, University of Lisbon, Lisbon, Portugal; Department of Chest Diseases, Çukuova University, School of Medicine, Adana, Turkey; Epidemiological Laboratory, Khartoum, Sudan; Department of Medicine, University of the West Indies, Mona Campus, Kingston, Jamaica; Department of Pulmonary Medicine, Paracelsus Medical University, Salzburg, Austria; Department of Pneumology, Hannover Medical School and German Centre of Lung Research, Hannover, Germany; University of British Columbia, Centre for Heart Lung Innovation, St Paul’s Hospital, Vancouver, BC, Canada; Division of Pulmonology and UCT Lung Institute, Department of Medicine, University of Cape Town, Cape Town, South Africa; Groote Schuur Hospital, Cape Town, South Africa; Department of Pulmonary Medicine, Sheri Kashmir Institute of Medical Sciences, Srinagar, Jammu and Kashmir, India; Department of International Health, John Hopkins Bloomberg School of Public Health, Baltimore, MD, USA; Ntational Heart and Lung Instiute, Imperial College London, London, UK; NIHR Imperial Biomedical Research Centre, London, UK; Oregon Health and Science University, Portland, OR, USA; Ntational Heart and Lung Instiute, Imperial College London, London, UK

**Keywords:** Chronic obstructive pulmonary disease, global health, non-communicable respiratory disease

Key Features

- The Burden of Obstructive Lung Disease (BOLD) study was established to assess the prevalence of chronic airflow obstruction, a key characteristic of chronic obstructive pulmonary disease, and its risk factors in adults (≥40 years) from general populations across the world.
- The baseline study was conducted between 2003 and 2016, in 41 sites across Africa, Asia, Europe, North America, the Caribbean and Oceania, and collected high-quality pre- and post-bronchodilator spirometry from 28 828 participants.
- The follow-up study was conducted between 2019 and 2021, in 18 sites across Africa, Asia, Europe and the Caribbean. At baseline, there were in these sites 12 502 participants with high-quality spirometry. A total of 6452 were followed up, with 5936 completing the study core questionnaire. Of these, 4044 also provided high-quality pre- and post-bronchodilator spirometry.
- On both occasions, the core questionnaire covered information on respiratory symptoms, doctor diagnoses, health care use, medication use and ealth status, as well as potential risk factors. Information on occupation, environmental exposures and diet was also collected.
- Collaborative research proposals and access to data requests should be submitted to Dr Andre F S Amaral [a.amaral@imperial.ac.uk]. For more information on the BOLD study, please visit [https://www.imperial.ac.uk/nhli/bold].

## Why was the cohort set up?

At the end of the 20th century, chronic obstructive pulmonary disease (COPD) was already considered a leading cause of morbidity and mortality.^1–3^ Yet, little was known about its prevalence and aetiology, particularly in low- and middle-income countries (LMICs). This information is important to improve the understanding of the impact of the disease on quality of life and health care cost, as well as to identify ways to reduce its risk.

The Burden of Obstructive Lung Disease (BOLD) study was set up and launched across several regions of the world as a network of population-based surveys using a standardized protocol. The initial main aims of the study were to assess the worldwide prevalence of chronic airflow obstruction, which is a defining characteristic of COPD, and to identify its main risk factors. The main aims of the follow-up study were: (i) to quantify the rate of lung function decline during adulthood; (ii) to assess the risk factors associated with lung function decline; and (iii) to understand the relationship between lung function and mortality, particularly across different ethnic groups.

The baseline study was funded in part by a grant from the Wellcome Trust (085790/Z/08/Z), which supported the coordinating centre in London, UK, and in part by unrestricted educational grants from University of Kentucky, Aventis, AstraZeneca, Boehringer-Ingelheim, Chiesi, GlaxoSmithKline, Merck, Novartis, Pfizer, Schering-Plough and Sepracor, which supported the initial coordinating centre in Portland, OR, USA. Additional support was provided to several sites in the baseline study (please see Funding for details). The follow-up study in LMICs was funded by the UK Medical Research Council (MR/R011192/1) and in European countries by AstraZeneca AB (ESR-17-13417).

## Who is in the cohort?

**Figure 1.**
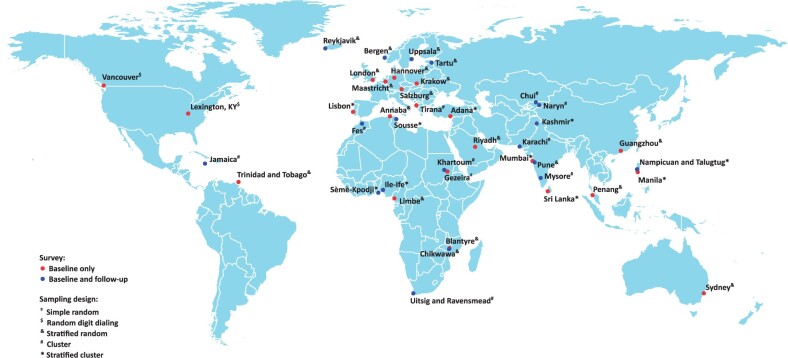
Burden of Obstructive Lung Disease (BOLD) study sites

The baseline study was conducted, between 2003 and 2016, in 41 sites in 34 countries across Africa, Asia, Europe, North America, the Caribbean and Oceania [https://www.imperial.ac.uk/nhli/bold] (Figure 1).^4^ These were selected to represent most of the regions covered by the Global Burden of Disease Programme,^5^ while over-representing larger regions such as South Asia and excluding Latin America, which had a separate study (PLATINO).^6^ Participants were non-institutionalized adults, aged 40 years and over, recruited from the general population around sites with at least 150 000 inhabitants. Sampling varied across sites, with some using simple random sampling and others using either stratified random sampling or cluster sampling (Figure 1). For each site and participant, weights were derived to account for sampling design.

At baseline, 77 640 contact attempts were made to recruit participants to the study, but 28 901 were ineligible (either died before clinic/home visit or left catchment area or were under 40 years old or were institutionalized or were untraceable or could not be contacted). Of the 48 739 eligible people, 14 482 (29.7%) were non-responders (actively refused to participate or provided partial data) and 34 257 (70.3%) were responders (completed the core questionnaire and post-bronchodilator spirometry, regardless of quality control score) (Table 1). Overall, at baseline the proportions of males and smokers were slightly higher among responders, and the proportion of people with a diagnosis of respiratory disease was slightly higher among non-responders. Non-responders were also older (Table 2).

**Table 1.** Numbers of contact attempts, ineligible people, non-responders, responders, response rate and cooperation rate in each Burden of Obstructive Lung Disease cohort site at baseline and at follow-up

	**Baseline** ^a^						**Follow-up** ^b^					
Site	Invites	Ineligible	Non-responders	Responders	Response rate, %	Cooperation rate, %	Invites	Ineligible (dead)	Non-responders	Responders	Response rate, %	Cooperation rate, %
Albania (Tirana)	1200	24	190	986	82	84	—	—	—	—	—	—
Algeria (Annaba)	969	0	52	917	95	95	—	—	—	—	—	—
Australia (Sydney)	2488	738	1165	585	24	33	—	—	—	—	—	—
Austria (Salzburg)	2200	192	659	1349	61	67	—	—	—	—	—	—
Benin (Sèmè-Kpodji)	900	22	28	850	94	97	706	84 (14)	505	117	17	19
Cameroon (Limbe)	719	24	262	433	60	62	—	—	—	—	—	—
Canada (Vancouver)	2434	659	919	856	35	48	—	—	—	—	—	—
China (Guangzhou)	690	0	88	602	87	87	—	—	—	—	—	—
England (London)	4467	2540	1230	697	16	36	—	—	—	—	—	—
Estonia (Tartu)	1440	482	300	658	46	69	619	219 (219)	1	399	64	100
Germany (Hannover)	2546	1092	741	713	28	49	—	—	—	—	—	—
Iceland (Reykjavik)	1000	94	146	760	76	84	757	260 (187)	119	378	50	76
India (Kashmir)	1100	17	130	953	87	88	771	660 (18)	22	89	12	80
India (Mumbai)	1857	1069	273	515	28	65	—	—	—	—	—	—
India (Mysore)	945	73	147	725	77	83	607	57 (23)	13	537	88	98
India (Pune)	1438	0	50	1388	97	97	851	146 (103)	11	694	82	98
Jamaica	907	19	93	795	88	90	594	356 (10)	141	97	16	41
Kyrgyzstan (Chui)	1226	151	5	1070	87	100	894	316 (42)	108	470	53	81
Kyrgyzstan (Naryn)	1202	92	5	1105	92	100	865	162 (47)	79	624	72	89
Malawi (Blantyre)	760	67	108	585	77	84	—	—	—	—	—	—
Malawi (Chikwawa)	982	153	1	828	84	100	448	69 (28)	5	374	85	99
Malaysia (Penang)	1218	407	98	713	59	88	—	—	—	—	—	—
Morocco (Fes)	985	0	19	966	98	98	770	571 (2)	134	65	9	33
Netherlands (Maastricht)	1341	198	513	630	47	55	—	—	—	—	—	—
Nigeria (Ile-Ife)	1704	504	52	1148	67	96	884	405 (56)	17	462	52	96
Norway (Bergen)	1130	133	290	707	63	71	661	198 (139)	165	298	45	64
Pakistan (Karachi)	3172	2116	4	1052	33	100	616	227 (51)	130	259	42	67
Philippines (Manila)	1397	1	478	918	66	66	—	—	—	—	—	—
Philippines (Nampicuan-Talugtug)	1177	28	158	991	84	86	727	198 (116)	57	472	65	89
Poland (Krakow)	769	39	127	603	78	83	—	—	—	—	—	—
Portugal (Lisbon)	7123	4348	2030	745	10	27	—	—	—	—	—	—
Saudi Arabia (Riyadh)	866	0	82	784	91	91	—	—	—	—	—	—
S. Africa (Uitsig & Ravensmead)	1378	110	377	891	65	70	—	—	—	—	—	—
Sri Lanka	1406	12	214	1180	84	85	—	—	—	—	—	—
Sudan (Gezeira)	1361	315	212	834	61	80	—	—	—	—	—	—
Sudan (Khartoum)	698	2	101	595	85	85	520	450 (2)	13	57	11	81
Sweden (Uppsala)	998	31	379	588	59	63	551	106 (52)	170	275	50	62
Trinidad & Tobago	1424	0	37	1387	97	97	—	—	—	—	—	—
Tunisia (Sousse)	799	17	65	717	90	92	661	332 (46)	60	269	41	82
Turkey (Adana)	2077	45	1157	875	42	43	—	—	—	—	—	—
USA (Lexington, KY)	15 147	13 087	1497	563	4	27	—	—	—	—	—	—

a At baseline: ineligible people were those who died before clinic/home visit, who left the catchment area, who were under 40 years old, who were institutionalized, who were untraceable or who could not be contacted. Non-responders were those who actively refused to participate and those who provided partial data. Responders were those who completed the core questionnaire and post-bronchodilator spirometry, regardless of quality control score. Response rate was defined as the number of responders divided by the number of invites or attempts to contact potential participants. Cooperation rate was defined as the number of responders divided by the number of responders plus the number of non-responders.

b At follow-up: invites (attempts to contact) were made to those who responded at baseline and had useable spirometry. Ineligible people were those who died between baseline and follow-up, who left the catchment area, who were untraceable or who could not be contacted. Non-responders were those who actively refused to participate and those who provided partial data. Responders were those who completed the core questionnaire. Response rate was defined as the number of responders divided by the number of invites or attempts to contact potential participants. Cooperation rate was defined as the number of responders divided by the number of responders plus the number of non-responders.

**Table 2.** Selected characteristics comparing non-responders and responders at baseline in the Burden of Obstructive Lung Disease cohort

	All 41 sites		18 sites with follow-up data	
	Non-responders (*n* = 14 482)	Responders (*n* = 34 257)	Non-responders (*n* = 1973)	Responders (*n* = 15 896)
Sex, %				
Male	44.3	46.7	46.0	45.2
Female	55.7	53.3	54.0	54.8
Age (years), mean (SD)	62 (15)	55 (11)	58 (14)	55 (12)
Ever smokers,^a^ %	35.2	37.9	34.0	32.1
Doctor-diagnosed asthma, emphysema, chronic bronchitis or chronic obstructive pulmonary disease,^b^ %	12.7	10.6	7.8	7.9
Other comorbid conditions,^c^ %	33.8	33.4	32.8	29.7

Sites names (*n *=* *41): Albania (Tirana); Algeria (Annaba); Australia (Sydney); Austria (Salzburg); **Benin (Sèmè-Kpodji)**; Cameroon (Limbe); Canada (Vancouver); China (Guangzhou); England (London); **Estonia (Tartu)**; Germany (Hannover); **Iceland (Reykjavik)**; **India (Kashmir)**; India (Mumbai); **India (Mysore)**; **India (Pune)**; **Jamaica**; **Kyrgyzstan (Chui)**; **Kyrgyzstan (Naryn)**; Malawi (Blantyre); **Malawi (Chikwawa)**; Malaysia (Penang); **Morocco (Fes)**; Netherlands (Maastricht); **Nigeria (Ile-Ife)**; **Norway (Bergen)**; **Pakistan (Karachi)**; Philippines (Manila); **Philippines (Nampicuan-Talugtug)**; Poland (Krakow); Portugal (Lisbon); Saudi Arabia (Riyadh); S. Africa (Uitsig & Ravensmead); Sri Lanka; Sudan (Gezeira); **Sudan (Khartoum)**; **Sweden (Uppsala)**; Trinidad & Tobago; **Tunisia (Sousse)**; Turkey (Adana); USA (Lexington, KY). Sites in bold type have follow-up data (*n *=* *18).

a Missing for 11 194 non-responders (41 sites); missing for 1164 non-responders (18 sites).

b Missing for 4487 non-responders (41 sites); missing for 767 non-responders (18 sites).

In total, high-quality post-bronchodilator spirometry data are available for 28 828 participants (52.6% females, 47.4% males). The mean age of participants was 55 years, the mean body mass index (BMI) was 26.7 kg/m^2^, 39.8% had ever smoked and 25.8% had higher education. The mean post-bronchodilator FVC (forced vital capacity) was 3.24 L and the mean post-bronchodilator FEV_1_ (forced expiratory volume in one second)/FVC was 77.7%. Table 3 shows the distribution of these characteristics by sex.

**Table 3.** Selected characteristics of the Burden of Obstructive Lung Disease cohort participants who completed the core questionnaire and had provided useable spirometry at baseline (41 sites; *n *=* *28 828)

Characteristic	Male (*n* = 13 659)	Female (*n* = 15 169)
Age (years), mean (SD)	55.1 (11.0)	54.2 (10.9)
Body mass index (kg/m^2^), mean (SD)	25.7 (4.9)	27.6 (7.1)
Ever smokers, %	59.2	22.4
Higher education, %	24.2	25.3
Post-bronchodilator FVC (L), mean (SD)	3.82 (0.98)	2.72 (0.71)
Post-bronchodilator FEV1/FVC (%), mean (SD)	76.4 (9.4)	78.8 (7.9)

FEV_1_ (forced expiratory volume in one second); FVC, forced vital capacity; SD, standard deviation.

## How often have they been followed up?

**Figure 2.**
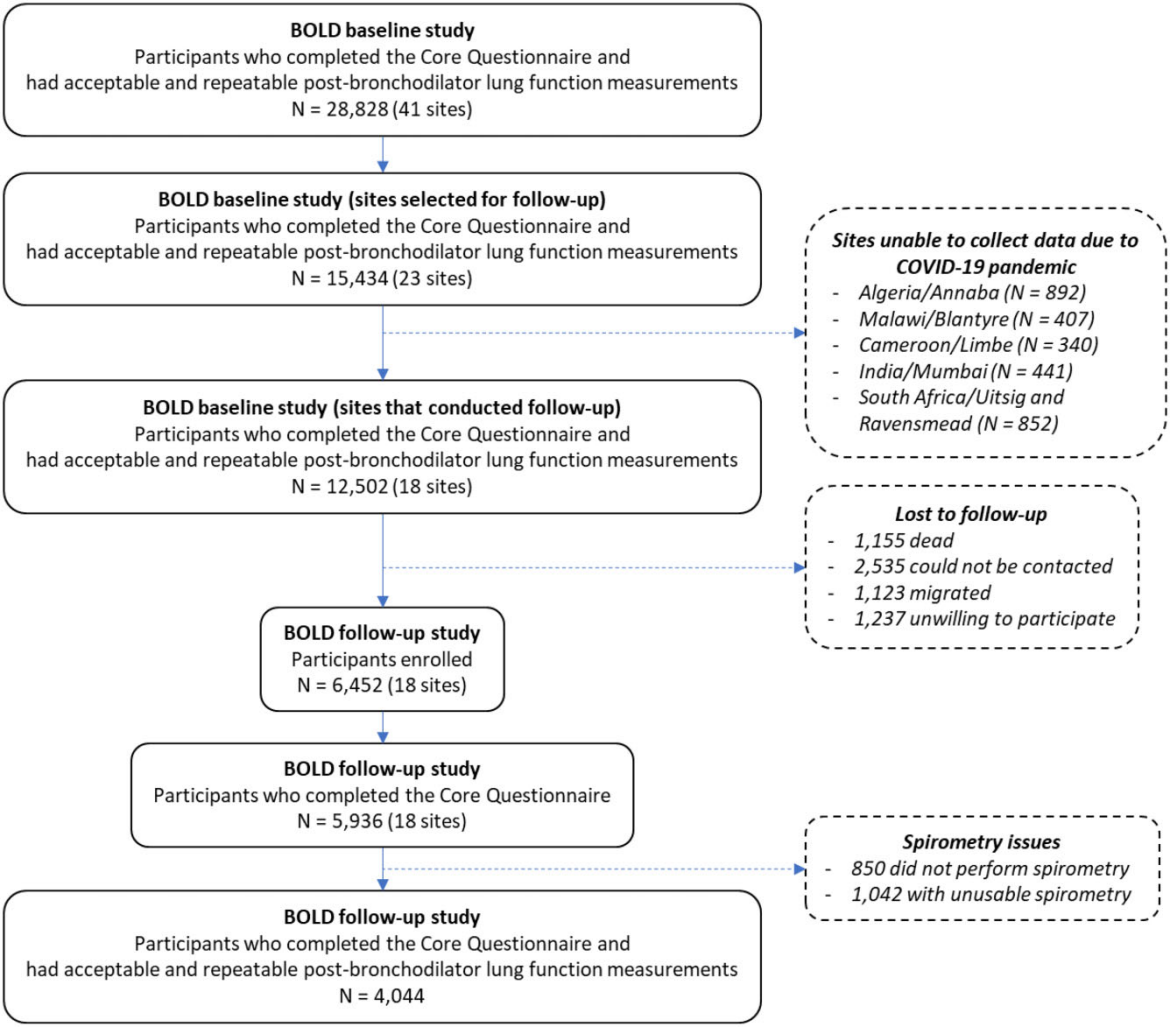
Selection of participants in the Burden of Obstructive Lung Disease (BOLD) cohort

The follow-up study was planned for 23 sites, but it was not feasible in five sites due to the COVID-19 pandemic. Participants from the BOLD baseline study were followed up once, between 2019 and 2021, in 14 sites in LMICs and four sites in Northern Europe, with a median follow-up time of 8.4 years. At follow-up, 12 502 individuals who had completed the core questionnaire, and had provided acceptable and repeatable lung function measurements at baseline, were invited to participate. The number of participants who completed the core questionnaire was 5936, and of these 4044 were able to perform spirometry and provided high-quality post-bronchodilator measurements for at least one lung function parameter (Figure 2). Slightly more than half of these participants were females (55.6%), the mean age was 61 years, the mean BMI was 26.5 kg/m^2^ and 30.7% had ever smoked. Table 4 shows the distribution of these characteristics by sex for participants who were followed up and had high-quality lung function data.

**Table 4.** Selected characteristics of the Burden of Obstructive Lung Disease cohort participants who completed the core questionnaire and had provided useable spirometry both at baseline and follow-up (*n *=* *4044)

	Baseline		Follow-up	
Characteristics	Male (*n* = 1802)	Female (*n* = 2242)	Male (*n* = 1802)	Female (*n* = 2242)
Age (years), mean (SD)	52.1 (9.3)	51.2 (9.1)	61.5 (10.1)	60.0 (10.0)
Body mass index (kg/m^2^), mean (SD)	25.0 (4.5)	26.1 (5.7)	25.7 (5.0)	27.2 (5.7)
Ever smokers, %	47.8	15.5	49.2	15.9
Higher education, %	27.6	31.6	27.6	31.6
Post-bronchodilator FVC (L), mean (SD)	3.87 (0.99)	2.73 (0.70)	3.54 (0.91)	2.51 (0.64)
Post-bronchodilator FEV1/FVC (%), mean (SD)	77.9 (7.5)	79.7 (6.9)	75.9 (8.3)	78.2 (7.1)

FEV_1_ (forced expiratory volume in one second); FVC, forced vital capacity; SD, standard deviation.

Among participants lost to follow-up, 1155 had died, 3658 had migrated or were unreachable and 1237 refused to participate. To explore reasons for not being able to participate in the follow-up, we investigated potential explanatory variables (sex, age, BMI, smoking status, education level, self-reported doctor diagnosis of cardiovascular disease, self-reported doctor diagnosis of diabetes, a history of tuberculosis, dyspnoea, chronic cough, chronic phlegm and wheezing) using logistic regression within each site and then pooling together estimates using random effects meta-analysis. Older participants, current smokers and those with lower BMI were more likely to be lost to follow-up (Table 5). Based on this information, we calculated inverse probability weights to correct for loss-to-follow up in future analyses.^7^^,^^8^

**Table 5.** Pooled odds ratio (OR) and 95% confidence interval (CI) of being lost to follow-up

Variable	**Adjusted OR (95% CI)** ^a^	Heterogeneity	
		I^2^ (%)	***P*** ^b^
Sex			
Male	(ref)	—	—
Female	1.03 (0.80–1.32)	78.8	<0.001
Age (years)	1.03 (1.01–1.04)	85.2	<0.001
Body mass index (kg/m^2^)	0.98 (0.97–0.99)	24.7	0.2
Smoking status			
Current	(ref)	—	—
Former	0.86 (0.65–1.13)	40.1	0.06
Never	0.75 (0.63–0.89)	13.4	0.4
Education level			
Tertiary	(ref)	—	—
Secondary	0.93 (0.82–1.07)	1.2	0.2
None to primary	1.05 (0.86–1.29)	36.2	0.06
Doctor diagnosis			
Cardiovascular disease	1.21 (0.92–1.58)	45.9	0.04
Diabetes	0.81 (0.56–1.19)	53.3	0.03
Tuberculosis	0.90 (0.55–1.48)	37.7	0.05
Respiratory symptoms			
Dyspnoea	0.94 (0.73–1.21)	46.7	0.02
Chronic cough	0.98 (0.75–1.23)	36.7	0.03
Chronic phlegm	0.89 (0.65–1.20)	44.6	0.009
Wheeze	1.02 (0.87–1.20)	0.0	0.8

a Adjusted simultaneously for all variables in the table.

b *P-*value from chi square test for heterogeneity across sites.

## What has been measured?

In both surveys, participants were asked to answer a set of standardized questionnaires and undergo a series of measurements. The questionnaires were translated into the local language of each site, back-translated to English and checked for accuracy before administration by trained staff. Questionnaires were developed to obtain information about respiratory symptoms, respiratory and cardiometabolic diagnoses, health care use, medication use, activity limitation and health status, as well as about potential risk factors, including tobacco smoking, occupational and environmental exposures and diet (Table 6). In addition, measurements of several anthropometric parameters, blood pressure and pulse rate (M2 Basic, Omron), and lung function were taken. Lung function testing was performed using a spirometer (EasyOne, ndd Medizintechnik AG), before and after the administration of 200 μg of salbutamol via an inhalation spacer (Able, Clement Clarke International). All spirometry curves were checked centrally at the BOLD Operations Centre, and to be considered useable, tests had to include at least three acceptable curves (no hesitation, complete blow, no artefact affecting lung function readings), with the two best blows being within 200 mL of each other. Prior to the start of the surveys, study site staff underwent a 1-week intensive training which covered consenting, questionnaire data collection, spirometry testing and quality control, anthropometry measurements and data transfer.

**Table 6.** Information collected in the Burden of Obstructive Lung Disease cohort at baseline and follow-up

Information	Baseline	Follow-up
Core questionnaire		
Smoking status/history	Yes	Yes
Respiratory symptoms (cough, sputum, wheezing, shortness of breath)	Yes	Yes
Respiratory diagnoses (asthma, emphysema, chronic bronchitis, COPD)	Yes	Yes
Cardiometabolic diagnoses (cardiovascular disease, hypertension, diabetes)	Yes	Yes
History of tuberculosis	Yes	Yes
Health care use	Yes	Yes
Medication use (respiratory)	Yes	Yes
Quality of life (mental and physical health)	Yes	Yes
Physical activity	No	Yes
Sleep	No	Yes
Occupational questionnaire		
High-risk jobs	Yes	Yes
Job history	No	Yes
Job exposure matrix linkage	No	Yes
Environmental questionnaire		
Solid fuel use	Yes	Yes
Pesticide use	No	Yes
Proximity to road	No	Yes
GPS coordinates (home)^a^	No	Yes
Food frequency questionnaire^a^	Yes	Yes
Physical measurements		
Spirometry	Pre- and post-bronchodilator	Pre- and post-bronchodilator
Anthropometry	Standing height, weight	Standing height, weight, ulna length, fibula length, waist circumference, hip circumference, neck circumference
Cardiovascular disease markers	No	Blood pressure, pulse rate

COPD, chronic obstructive pulmonary disease; GPS, global positioning system.

a Some sites only.

## What has it found? Key findings and publications

BOLD has published extensively on the prevalence and aetiology of chronic airflow obstruction. By April 2023, there were 102 publications in peer-reviewed journals [https://www.imperial.ac.uk/nhli/bold/publications]. Here we highlight the main findings from this study to date.

- The prevalence of chronic airflow obstruction varies widely across world regions but is, on average, slightly lower in LMICs and more common among males (11.2%) than among females (8.6%). Among males, the prevalence of chronic airflow obstruction ranges from 3.5% in Riyadh (Saudi Arabia) to 23.2% in Uitsig and Ravensmead (South Africa), and among females from 2% in Sousse (Tunisia) to 19.4% in Salzburg (Austria)^4^; The main risk factors for chronic airflow obstruction are tobacco smoking (both active and passive), which accounts for approximately 46% of the prevalence in males and 26% in females. The next most important risk factors are a poor education level and poverty, followed by a history of tuberculosis (where tuberculosis is common), a low BMI and exposure to dust in the workplace for more than 10 years.^4,^^9–12^
- Ambient particulate matter and the use of solid fuels for cooking and heating are unlikely to explain a substantial amount of the prevalence of chronic airflow obstruction. These findings are equally true for males and females.^13^^,^^14^
- The prevalence of small airways obstruction in the presence of what is usually considered normal lung function (i.e. isolated small airways obstruction) is common in general populations across the world. In addition, the main risk factors for isolated small airways obstruction are the same as for chronic airflow obstruction, suggesting that the former has the potential to predict the latter.^15^
- There is a large proportion of people with low forced vital capacity (FVC), suggestive of low lung volumes, in LMICs, particularly in sub-Saharan Africa. In addition, this low FVC is associated with cardiometabolic diseases (i.e. cardiovascular disease, hypertension and diabetes)^16^ and cardiometabolic risk factors.^17^

## What are the main strengths and weaknesses?

The BOLD study has brought together a strong and widespread international collaboration to address the epidemiology of COPD and is today a reference in the field of chronic respiratory diseases. In addition, this study has had an important impact on capacity building and promotion of equity and inclusion across the study sites.

The main strengths of the BOLD study are the:

- broad coverage of world regions and ethnic groups;
- large sample of representative population-based data;
- use of a standardized protocol, including the same questionnaires and same model of spirometers to test lung function, across study sites;
- centralized training and certification of interviewers and spirometry technicians. The quality of the data was monitored throughout the study, and re-training of staff was carried out if necessary;
- high quality of pre- and post-bronchodilator lung function measurements, with centralized quality control and assessment of all spirometry curves. In the follow-up study, we also developed and implemented an algorithm to monitor lung function data collection and provide feedback to spirometry technicians in ‘real-time’.

The main weaknesses are the:

- self-reported doctor diagnoses of COPD, asthma, heart disease, hypertension and diabetes, which are subject to recall bias and local diagnostic guidelines and patterns;
- limited data on causes of death in sites located in LMICs;
- except for four sites in Northern Europe, the lack of biological samples, including DNA samples, for investigation of biological mechanisms. These will be sought in the next wave of the study.

To compensate for non-response at baseline and at follow-up, we derived weights for respondents to the core questionnaire. However, we cannot dismiss the possibility of bias in our estimates due to unmeasured factors.

## Can I get hold of the data? Where can I find out more?

The study data are not freely accessible. However, proposals for collaboration will be considered. Requests should be directed to the project lead, Dr Andre F S Amaral [a.amaral@imperial.ac.uk].

## BOLD (Burden of Obstructive Lung Disease) Collaborative Research Group members

**Albania**: Hasan Hafizi (principal investigator [PI]), Anila Aliko, Donika Bardhi, Holta Tafa, Natasha Thanasi, Arian Mezini, Alma Teferici, Dafina Todri, Jolanda Nikolla, and Rezarta Kazasi (Tirana University Hospital Shefqet Ndroqi, Albania); **Algeria**: Hamid Hacene Cherkaski (PI), Amira Bengrait, Tabarek Haddad, Ibtissem Zgaoula, Maamar Ghit, Abdelhamid Roubhia, Soumaya Boudra, Feryal Atoui, Randa Yakoubi, Rachid Benali (Department of Pneumology, Faculty of Medicine, Annaba, Algeria), Abdelghani Bencheikh and Nadia Ait-Khaled (Department of Epidemiology and Prevention, EPHS ElHadjar, Algeria); **Australia**: Christine Jenkins (PI), Guy Marks (PI), Tessa Bird, Paola Espinel, Kate Hardaker, Brett Toelle (Woolcock Institute of Medical Research, Sidney, Australia); **Austria**: Michael Studnicka (PI), Torkil Dawes, Bernd Lamprecht, and Lea Schirhofer (Department of Pulmonary Medicine, Paracelsus Medical University, Salzburg, Austria); **Benin**: Herve Lawin (PI), Arsene Kpangon, Karl Kpossou, Gildas Agodokpessi, Paul Ayelo, Benjamin Fayomi, Rolus Atrokpo, Gaston Hounton, Dieudonnè Yadjodo (Unit of Teaching and Research in Occupational and Environmental Health, University of Abomey Calavi, Cotonou, Benin); **Cameroon**: Bertrand Mbatchou (PI), Atongno Humphrey Ashu (Douala General Hospital, Douala, Cameroon); **Canada**: Wan C Tan (PI) and Wen Wang (iCapture Center for Cardiovascular and Pulmonary Research, University of British Columbia, Vancouver, BC, Canada); **China**: NanShan Zhong (Principal Investigator [PI]), Shengming Liu, Jiachun Lu, Pixin Ran, Dali Wang, Jin-ping Zheng, and Yumin Zhou (Guangzhou Institute of Respiratory Health, First Affiliated Hospital of Guangzhou Medical College, Guangzhou, China); **Estonia**: Rain Jõgi (PI), Hendrik Laja, Katrin Ulst, Vappu Zobel, Toomas-Julius Lill, Katrin Kiili, and Ira Laanelepp (Lung Clinic, Tartu University Hospital, Tartu, Estonia); **Germany**: Tobias Welte (PI), Isabelle Bodemann, Henning Geldmacher, and Alexandra Schweda-Linow (Dept of Pneumology, Hannover Medical School and German Center of Lung Research, Hannover, Germany); **Iceland**: Thorarinn Gislason (PI), Bryndis Benedikdtsdottir, Kristin Jörundsdottir, Lovisa Gudmundsdottir, Sigrun Gudmundsdottir, Gunnar Gudmundsson, Elin Helga Thorarinsdottir, and Hjördis Sigrun Pálsdottir (Department of Allergy, Respiratory Medicine, and Sleep, Landspitali University Hospital, Reykjavik, Iceland); **India**: Mahesh Padukudru Anand (PI) (JSS Medical College, JSSAHER, Mysuru, India); Parvaiz A Koul (PI), Sajjad Malik, Nissar A Hakim, and Umar Hafiz Khan (Sher-i-Kashmir Institute of Medical Sciences, Srinagar, J&K, India); Rohini Chowgule (PI), Vasant Shetye, Jonelle Raphael, Rosel Almeda, Mahesh Tawde, Rafiq Tadvi, Sunil Katkar, Milind Kadam, Rupesh Dhanawade, and Umesh Ghurup (Indian Institute of Environmental Medicine, Mumbai, India); Sanjay Juvekar (PI), Siddhi Hirve, Somnath Sambhudas, Bharat Chaidhary, Meera Tambe, Savita Pingale, Arati Umap, Archana Umap, Nitin Shelar, Sampada Devchakke, Sharda Chaudhary, Suvarna Bondre, Savita Walke, Ashleshsa Gawhane, Anil Sapkal, Rupali Argade, Vijay Gaikwad, Dhiraj Agrawal, Babu Pawar, Shalan Mhetre, Namdev Kale, and Shirish Kathale (Vadu Rural Health Program, Pune, India); Sundeep Salvi (PI), Bill Brashier, Jyoti Londhe, and Sapna Madas (Chest Research Foundation, Pune, India); **Jamaica**: Althea Aquart-Stewart (PI), Akosua Francia Aikman (University of the West Indies, Kingston, Jamaica); **Kyrgyzstan**: Talant M Sooronbaev (PI), Bermet M Estebesova, Meerim Akmatalieva, Saadat Usenbaeva, Jypara Kydyrova, Eliza Bostonova, Ulan Sheraliev, Nuridin Marajapov, Nurgul Toktogulova, Berik Emilov, Toktogul Azilova, Gulnara Beishekeeva, Nasyikat Dononbaeva, and AijamalTabyshova (Pulmunology and Allergology Department, National Centre of Cardiology and Internal Medicine, Bishkek, Kyrgyzstan); **Malawi**: Kevin Mortimer (Baseline PI), Wezzie Nyapigoti, Ernest Mwangoka, Mayamiko Kambwili, Martha Chipeta, Gloria Banda, Suzgo Mkandawire, Justice Banda, Graham Devereux (Follow-up PI), Jamie Rylance, Martin Njoroge, Catherine Chirwa, Chifundo Mhango, Edgar Ngwira, Faith Zumazuma, Frank Jonas, and Patrick Mjojo (the Malawi Liverpool Wellcome Trust, Blantyre, Malawi); **Malaysia**: Li-Cher Loh (PI), Abdul Rashid, and Siti Sholehah (Royal College of Surgeons in Ireland and University College Dublin Malaysia Campus (RUMC)); **Morocco**: Mohamed C Benjelloun (Baseline PI), Chakib Nejjari, Mohamed Elbiaze, Karima El Rhazi (Follow-up PI), Manelle Rjimati, Btissame ElHarche, Reda Benjelloun, and Yassin Chefchaou (Laboratoire d’épidémiologie, Recherche Clinique et Santé Communautaire, Fès, Morroco); **The Netherlands**: E F M Wouters and G J Wesseling (Maastricht University Medical Center, Maastricht, the Netherlands); **Nigeria**: Daniel Obaseki (PI), Gregory Erhabor, Olayemi Awopeju, and Olufemi Adewole (Obafemi Awolowo University, Ile-Ife, Nigeria); **Norway**: Amund Gulsvik (Baseline PI), Tina Endresen, Lene Svendsen (Department of Thoracic Medicine, Institute of Medicine, University of Bergen, Bergen, Norway), and Rune Nielsen (Follow-up PI), Marit Aardal, Hildegunn B Fleten, Gerd Eli Dale, Eli Nordeide, Malin P Grøttveit, Åsa Skjelde, Ane Aamli Gagnat, Anders Ørskov Rotevatn, Marta Erdal (Department of Clinical Science, University of Bergen, Bergen, Norway); **Pakistan**: Asaad A Nafees (PI), Muhammad Irfan, Hasan Nawaz Tahir, Muhammad Noman, Roman Ul Haq (Aga Khan Univeristy, Karachi, Pakistan); **Philippines**: Luisito F Idolor (Baseline PI), Teresita S de Guia, Norberto A Francisco, Camilo C Roa, Fernando G Ayuyao, Cecil Z Tady, Daniel T Tan, Sylvia Banal-Yang, Vincent M Balanag, Jr, Maria Teresita N Reyes, Renato B Dantes, and Stefanni Nonna M Paraguas (Follow-up PI) (Lung Centre of the Philippines and Philippine Heart Centre, Philippine General Hospital, Nampicuan and Talugtug, the Philippines); Renato B Dantes (Baseline PI), Lourdes Amarillo, Lakan U Berratio, Lenora C Fernandez, Norberto A Francisco, Gerard S Garcia, Teresita S de Guia, Luisito F Idolor, Sullian S Naval, Thessa Reyes, Camilo C Roa, Jr, Ma Flordeliza Sanchez, and Leander P Simpao (Philippine College of Chest Physicians, Manila, the Philippines); **Poland**: Ewa Nizankowska-Mogilnicka (PI), Jakub Frey, Rafal Harat, Filip Mejza, Pawel Nastalek, Andrzej Pajak, Wojciech Skucha, Andrzej Szczeklik, and Magda Twardowska, (Division of Pulmonary Diseases, Department of Medicine, Jagiellonian University School of Medicine, Krakow, Poland); **Portugal**: Cristina Bárbara (PI), Fátima Rodrigues, Hermínia Dias, João Cardoso, João Almeida, Maria João Matos, Paula Simão, Moutinho Santos, and Reis Ferreira (the Portuguese Society of Pneumology, Lisbon, Portugal); **Saudi Arabia**: M Al Ghobain (PI), H Alorainy (PI), E El-Hamad, M Al Hajjaj, A Hashi, R Dela, R Fanuncio, E Doloriel, I Marciano, and L Safia (Saudi Thoracic Society, Riyadh, Saudi Arabia); **South Africa**: Eric Bateman (Baseline PI), Anamika Jithoo (Baseline PI), Desiree Adams, Edward Barnes, Jasper Freeman, Anton Hayes, Sipho Hlengwa, Christine Johannisen, Mariana Koopman, Innocentia Louw, Ina Ludick, Alta Olckers, Johanna Ryck, Janita Storbeck, and Richard van Zyl-Smit (Follow-up PI) (University of Cape Town Lung Institute, Cape Town, South Africa); **Sri Lanka**: Kirthi Gunasekera (PI), Rajitha Wickremasinghe (Medical Research Institute, Central Chest Clinic, Colombo, Sri Lanka); **Sudan**: Asma Elsony (Baseline PI), Hana A Elsadig, Nada Bakery Osman, Bandar Salah Noory, Monjda Awad Mohamed, Hasab Alrasoul Akasha Ahmed Osman, Namarig Moham ed Elhassan, Abdel Mu‘is El Zain, Marwa Mohamed Mohamaden, Suhaiba Khalifa, Mahmoud Elhadi, Mohand Hassan, Dalia Abdelmonam, Rana Ahmed (Follow-up PI), Rashid Osman, Hind Eltigani, Najlaa Mohamed Abass, Ahmed Beriar Ahmed, Sahar AlaElddin (Epidemiological Laboratory, Khartoum, Sudan); **Sweden**: Christer Janson (PI), Inga Sif Olafsdottir, Katarina Nisser, Ulrike Spetz-Nyström, Gunilla Hägg, Gun-Marie Lund, Andrei Malinovschi, Eva Wallberg, Birgitta Appelfeldt, and Mona Andrén (Department of Medical Sciences: Respiratory Medicine and Allergology, Uppsala University, Uppsala, Sweden); **Trinidad and Tobago**: Terence Seemungal (PI), Fallon Lutchmansingh, Liane Conyette (University of the West Indies, St Augustine, Trinidad and Tobago); **Tunisia**: Imed Harrabi (Baseline PI), Myriam Denguezli (Follow-up PI), Zouhair Tabka (deceased), Hager Daldoul, Zaki Boukheroufa, Firas Chouikha, Wahbi Belhaj Khalifa, Safa Hsan, Nadia Lakhdar, and Mounir Landolsi (University Hospital Farhat Hached, Faculté de Médecine, Sousse, Tunisia); **Turkey**: Ali Kocabaş (PI), Attila Hancioglu, Ismail Hanta, Sedat Kuleci, Ahmet Sinan Turkyilmaz, Sema Umut, and Turgay Unalan (Department of Chest Diseases, Cukurova University School of Medicine, Adana, Turkey); **UK**: Peter G J Burney (Baseline and Follow-up PI), Anamika Jithoo, Louisa Gnatiuc, Hadia Azar, Jaymini Patel, Caron Amor, James Potts, Michael Tumilty, Fiona McLean, Risha Dudhaiya, Andre F S Amaral (Project lead), Octavia Mulhern, Emmanouil Bagkeris, Jasleen Gegic, Paul Cullinan, Cosetta Minelli (National Heart and Lung Institute, Imperial College London, London, UK); **USA**: A Sonia Buist (Baseline PI) (Oregon Health & Science University, Portland, OR), Mary Ann McBurnie, William M Vollmer, Suzanne Gillespie (Kaiser Permanente Center for Health Research, Portland, OR); Sean Sullivan (University of Washington, Seattle, WA); Todd A Lee, Kevin B Weiss, (Northwestern University, Chicago, IL); Robert L Jensen, Robert Crapo (Latter Day Saints Hospital, Salt Lake City, Utah); Paul Enright (University of Arizona, Tucson, AZ); David M Mannino (PI), John Cain, Rebecca Copeland, Dana Hazen, and Jennifer Methvin, (University of Kentucky, Lexington, KY); Vanessa Garcia Larsen (John Hopkins Bloomberg School of Public Health, Baltimore, MD)

## Ethics approval

All sites received approval from their local ethics committee, the follow-up study was also approved by Imperial College London Research Ethics Committee (ref. 17IC4272), and participants provided informed consent.

## Data Availability

See Can I get hold of the data? above.
